# Importance of phenotypic plasticity in crop resilience

**DOI:** 10.1093/jxb/erad465

**Published:** 2024-02-02

**Authors:** Roosa A E Laitinen

**Affiliations:** Organismal and Evolutionary Research Programme, Faculty of Biological and Environmental Sciences, Viikki Plant Science Centre, University of Helsinki, Helsinki, Finland

**Keywords:** Flowering time, G×E, genomic prediction, phenotypic plasticity, resilience

## Abstract

This article comments on:

Guo T, Wei J, Li X, Yu J. 2024. Environmental context of phenotypic plasticity in flowering time in sorghum and rice. Journal of Experimental Botany 75, 1004–1015.

**To achieve resilience in future agriculture, crops that can mitigate the effects of changing and unpredictable climate patterns are needed. Phenotypic plasticity allows plants to quickly respond to environmental changes, but plasticity as a trait is not often integrated into genomic prediction models. To utilize the full potential of plasticity in improving genomic predictions, the genetic and environmental factors underlying plasticity must be understood. Guo *et al*. (2024) investigated the influence of the number of different environments and the range of flowering time across genotypes of multiple field trials on the plasticity of flowering time in sorghum and rice. Their study provides valuable insights into the impact of environmental factors on accurate prediction of the genotype-by-environment interaction (G×E) effect on flowering time in these species. Understanding phenotypic plasticity and the factors influencing plant adaptation to future environments helps to improve the desired stability in crop production.**

Phenotypic plasticity refers to an organism’s ability to adjust its phenotype in response to different environments (Bradshaw, 1965). For immobile plants, phenotypic plasticity offers fast ways to optimize growth and development under changing conditions. Plasticity in flowering time is a notable example, preventing plants from flowering in conditions that would be detrimental to their survival. Phenotypic plasticity in response to an environmental cue is a trait itself and depends on the environment. It is controlled by both genetic and environmental factors and varies across species and traits. Genetic variation in phenotypic plasticity is often attributed to G×E (Fig. 1) (Schlichting, 1986; Pigliucci, 2005; Laitinen and Nikoloski, 2019; Napier *et al.*, 2023). G×E leads to challenges in the predictability and accuracy of genomic prediction models that often only capture trait mean values, thus compromising plant breeding efforts. Consequently, integrating phenotypic plasticity of focal traits in the form of G×E to genomic prediction models would enhance the predictability of unknown genotypes in future conditions (Tong *et al.*, 2023).

**Fig. 1.**
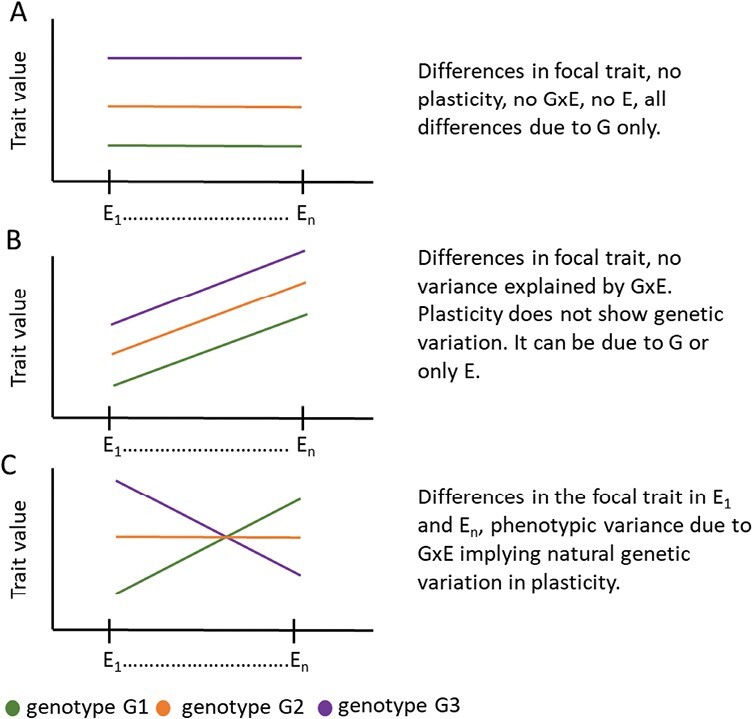
Relationship between plasticity and G×E. Graphs illustrating the three main scenarios of linear responses of three different genotypes in multiple environments. (A) Differences among genotypes in a trait in different environments do not always imply plasticity. (B) A trait can show plasticity, due either to the environment or to the same genetic factor(s), resulting in the same response across genotypes and lack of G×E. (C) The presence of G×E always implies genetic variation for phenotypic plasticity in the trait.

## Phenotypic plasticity across multiple field trials

Typically, plant responses are studied by examining the effects of a single environmental factor or treatment. However, this approach fails to account for the combined effect of multiple environmental cues that crop plants encounter in the field. To capture these cues, multi-environment field trials are commonly conducted to evaluate the performance of genotypes in different agricultural regions. Although evaluating G×E effects provides information of how traits are affected by the different environments within a population, it does not allow prediction of plasticity for individual genotypes.

Genotype-specific plasticity in response to different environments can be captured using linear regression of the reaction norms (Morrissey and Liefting, 2016), where two parameters, slope (representing plasticity) and intercept (representing genotypic value), of the regression are used. In maize, analysis of plasticity over 23 traits in response to at least four different environments has shown that trait plasticity has a separate genetic control from the mean value of the trait (Kusmec *et al.*, 2017). This validates the potential of specific breeding of desired plasticity without influencing the mean values of the focal traits (Kusmec *et al*. 2017).

In recent years, research has increasingly focused on the genetic patterns underlying plasticity of field-grown crops such as maize, rice, sorghum, wheat, oat, barley, and soybean (Kusmec *et al.*, 2017; Li *et al*., 2021; Liu *et al.*, 2021; Onogi *et al.*, 2021; Mu *et al.*, 2022; Waters *et al.*, 2023). A large-scale study involving a total of 488 maternal lines crossed with two elite paternal lines, evaluating 12 different traits in maize hybrids across 6–11 different environments, revealed dominant inheritance in plasticity of many agriculturally relevant traits (Liu *et al.*, 2021). Further quantification of linear plasticity showed that more of the phenotypic variance was explained by G×E than by the genotype alone. Moreover, the degree of plasticity was specific to a trait and varied across genotypes. These studies provide evidence that to develop strategies for future crop breeding, multi-environment field trials that aim at a detailed understanding of genetic and environmental factors controlling plasticity in crop plants are required.

## Environment number and range matter for accuracy of G×E prediction

Guo *et al*. (2024) conducted a study to test how the environmental sample size and the range of the environmental means affect the accuracy of the estimation of the plasticity of unknown genotypes in untested conditions. Environmental mean denotes the mean trait value of the genotypes across the studied environments. The authors used flowering time plasticity data from nine field experiments of sorghum and rice. The same datasets have previously been utilized to assess the genetic basis of plasticity in sorghum and rice (Li *et al.*, 2018; Guo *et al.*, 2020; Mu *et al.*, 2022). However, these studies did not focus on the contribution of the environment to the extent of plasticity. Guo *et al*. (2024) were able to gain information about the influence of environment on plasticity and genomic predictions.

Using reaction norms as a measure for plasticity, Guo *et al*. (2024) demonstrated that increasing both sample size and sample mean range of environments improved accuracy when predicting plasticity. Interestingly, their study also revealed that phenotypic plasticity could be estimated with a limited number of environments, if the environmental mean range was large enough. In addition, it was predicted that specific field sites were more prone to generate a larger environmental range than others. Accurate predictions were achieved based on the most extreme conditions that capture a significant portion of the variation in plasticity. Furthermore, the environment had stronger effects on the reaction norm slope than the intercept, probably reflecting the central role of the environment in plasticity.

**Fig. 2.**
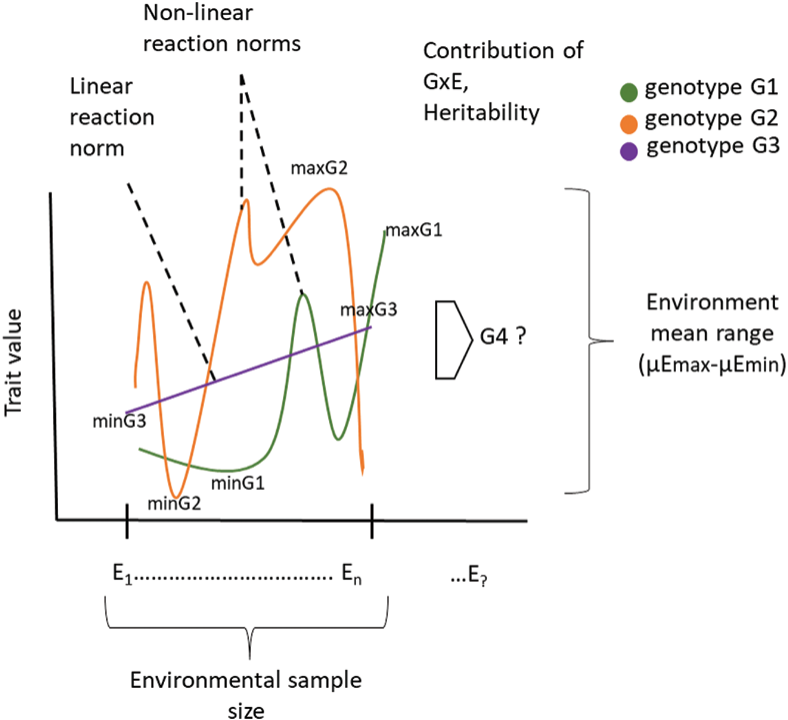
Factors contributing to the prediction of plasticity. Guo *et al*. (2024) have shown that environmental sample size and environmental mean (calculated as the average of the genotypes across environments) range affect the slope and intercept of the prediction norms and influence the prediction of plasticity. Genetic factors influencing prediction are the genetic variation or contribution of G×E and the heritability of the plasticity. In addition, the prediction of plasticity is strongly influenced by the choice of the quantification method of the reaction norms and the modelling approach. In the field, reaction norms exhibit both linear and non-linear plasticity patterns of different traits and vary across traits and environments.

This study provides valuable insights for designing field trials, suggesting that a limited number of environments can be informative if they cover large proportions of the environmental range. However, to date this finding was limited to linear trait plasticity, such as flowering time. One challenge when predicting behaviour in field conditions is that reaction norms can exhibit both linear and non-linear plasticity patterns of different traits (Fig. 2). It has been shown that for agricultural traits, non-linear responses are more common than linear responses for genotypes, with maximum and minimum performance not necessarily occurring at the extremes of the environmental range (Kusmec *et al.*, 2017). The proportions of linear or non-linear plasticity vary across different traits and environments and often the same trait exhibits both linear and non-linear plasticity depending on the specific environment (Kusmec *et al.*, 2017). Under field conditions, these patterns are likely to occur simultaneously. To ensure accurate predictions without prior knowledge of the environmental range, multi-environment trials can significantly improve accuracy when predicting plasticity in untested future conditions. Future research is needed to explore to what extent this also holds true for non-linear plasticity.

## Future challenges

Integration of plasticity in genomic prediction models requires understanding of the contribution of the different factors influencing plasticity over multiple environments (Fig. 2). In the end, all plant breeding relies on the genetic potential of the cultivars. This genetic potential can directly influence traits, or it may remain hidden and only become evident through its interaction with the environment (G×E), resulting in phenotypic plasticity. The applications in agriculture of understanding the genetic and environmental factors that control plasticity are twofold. First, it opens up new possibilities for plant breeding, providing a foundation to improve the stability of crops and for future environments. Second, increased plasticity can enhance genetic diversity in breeding populations.

To ensure accurate genomic predictions that can be applied for future field conditions, interdisciplinary collaborations that bridge the gap between greenhouse and field trials are required. These collaborations should aim to integrate modern data science methods to analyse and integrate data across different environments. As an example, recently a genome-scale metabolic network-based framework for genomic prediction, called netGSA, was developed, allowing integration of metabolomics data (Tong *et al.*, 2020). This framework was recently extended to predict plasticity in growth in *Arabidopsis thaliana* (Tong *et al.*, 2023). In addition, studies under controlled conditions with model species are required to detail the mechanisms that underlie trait plasticity. Nevertheless, these studies should be complemented with field trials to gain a full understanding of how the combined effects of different environmental factors influence traits over time.

The relevance of G×E for phenotypic variance and the potential of plasticity in plant adaptation and plant breeding is not in doubt, yet there are still several open questions in this field. What is the genetic architecture underlying different trait plasticities and can this be translated across species? What are the trade-offs associated with increased yield stability of agricultural crops? Are certain environments more dominant than others in terms of their influence on trait expression and plasticity, particularly those with larger ranges? What are the perspectives of modern imaging methods and growing conditions for precise phenotyping to improving predictability? How can we integrate the meteorological data to the modelling of crop behaviour? Pinpointing the contribution of genetic and environmental factors in different species will reveal the full potential of plasticity in yield stability breeding and will provide exciting avenues for further research.
